# A case of PD-L1 negative advanced pulmonary sarcomatoid carcinoma effectively treated with atezolizumab, carboplatin, paclitaxel, and bevacizumab

**DOI:** 10.1016/j.rmcr.2022.101579

**Published:** 2022-01-04

**Authors:** Kosuke Sawatari, Motohiro Izumi, Risa Sone, Tsuyoshi Hattori, Akira Sugimoto, Yosuke Eguchi, Takashi Mamoto

**Affiliations:** a Bell Land General Hospital, Department of Respiratory Medicine, 500-3, Higashiyama, Naka-ku, Sakai-city, Osaka, Japan; b Osaka City General Hospital, Department of Respiratory Medicine, 2-13-2, Miyakojima Hon-dori, Miyakojima-ku, Osaka-city, Osaka, Japan

**Keywords:** Pulmonary sarcomatoid carcinoma, PD-L1, Immune checkpoint inhibitor, Bevacizumab, Atezolizumab, Lung cancer

## Abstract

Pulmonary sarcomatoid carcinoma (PSC) is an extremely rare neoplasm with poor prognosis and no established treatment. A 50-year-old man presented with fever, was found to have a mass measuring 14 cm in the right upper lobe of the chest, along with right pleural effusion on computed tomography (CT). Positron emission tomography-CT revealed abnormal tracer uptake in the area corresponding to the mass in the upper lobe. Hence, convex-probe endobronchial ultrasound-guided transbronchial needle aspiration was performed. Histological examination revealed dense proliferation of spindle tumor cells and no programmed death-ligand 1 (PD-L1) expression. Thus, he was diagnosed with PSC (cT4N0M1a, clinical stage IVA), and four-agent combination chemotherapy with atezolizumab, carboplatin, paclitaxel, and bevacizumab was initiated. Marked shrinkage of the mass and symptomatic improvements were observed following the treatment initiation. Tumor shrinkage was further noted after shifting to maintenance therapy with atezolizumab and bevacizumab; the patient exhibited no symptom exacerbation 2 years later and continued the treatment. Our case showed that four-agent combination chemotherapy with atezolizumab, carboplatin, paclitaxel, and bevacizumab could be an effective treatment option for advanced PSC with or without PD-L1 expression.

## 1 Introduction

Pulmonary sarcomatoid carcinoma (PSC) is an extremely rare neoplasm, occurring in 0.4% of non-small cell lung cancer (NSCLC) cases [1], and exhibits poor prognosis with no established treatment. Recently, the programmed death-ligand 1 (PD-L1) expression rate has been reported to be higher in cases of PSC than those in other NSCLCs [2]. In addition, immune checkpoint inhibitors (ICIs) have been shown to be effective on PSCs with PD-L1 expression based on few case reports at present [3]; hence, further accumulation of cases is awaited. There is no report revealing the effectiveness of chemotherapy, including ICI, in PSC with no PD-L1 expression. We have reported a rare case of PSC with no PD-L1 expression, and it was successfully treated with four-agent combination chemotherapy, including an ICI and antiangiogenic therapy.

## 2 Case presentation

**Fig. 1:**
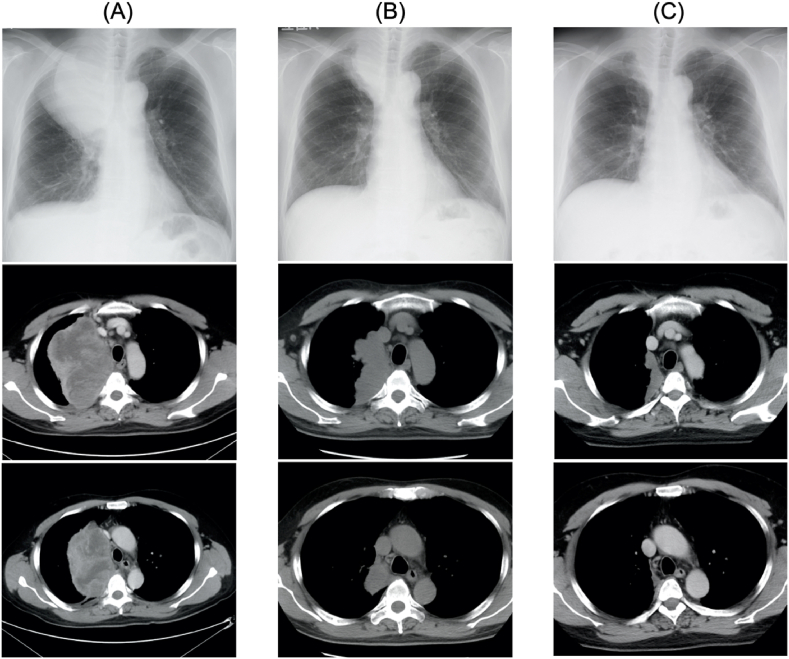
(A) Chest radiograph and chest CT images on admission. Chest radiograph image on admission shows a tumor shadow in the right upper lung field. Chest CT images reveal a huge tumor in the right upper lobe, invading the mediastinum and thoracic wall, and right small pleural effusion. The density of the tumor is irregular. (B) Five months after the initiation of therapy, the chest radiograph shows a noticeable reduction of the tumor shadow in the right upper lung field. Chest CT shows evident reduction of the tumor size in the right upper lobe, no significant mediastinal lymphadenopathy, and no pleural effusion. (C) Two years after the initiation of therapy: tumor shrinkage persists.

**Fig. 2:**
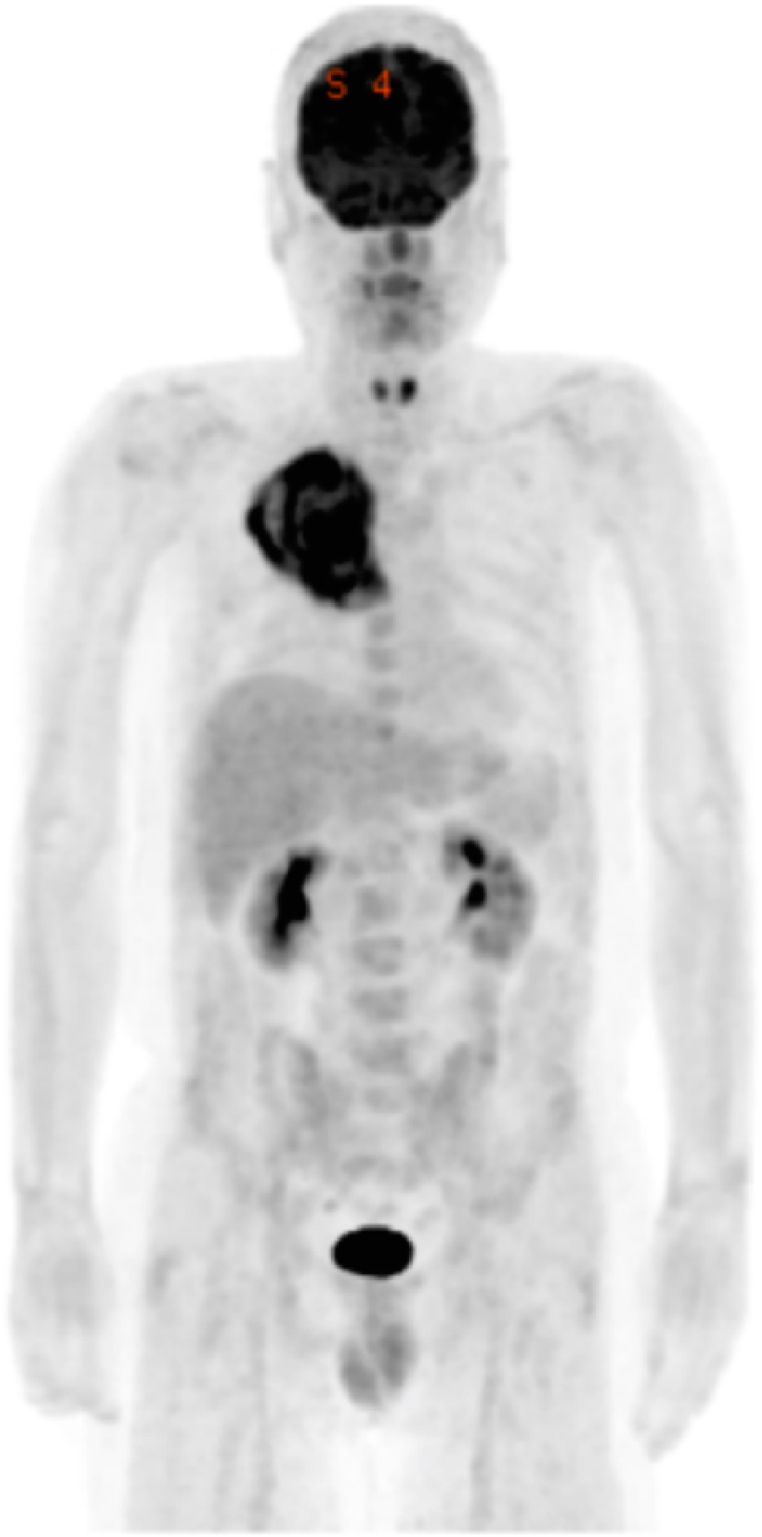
Positron emission tomography (PET) with 18F-fluorodeoxyglucose (FDG) shows the accumulation of the tracer in a tumor in the right upper lobe.

**Fig. 3:**
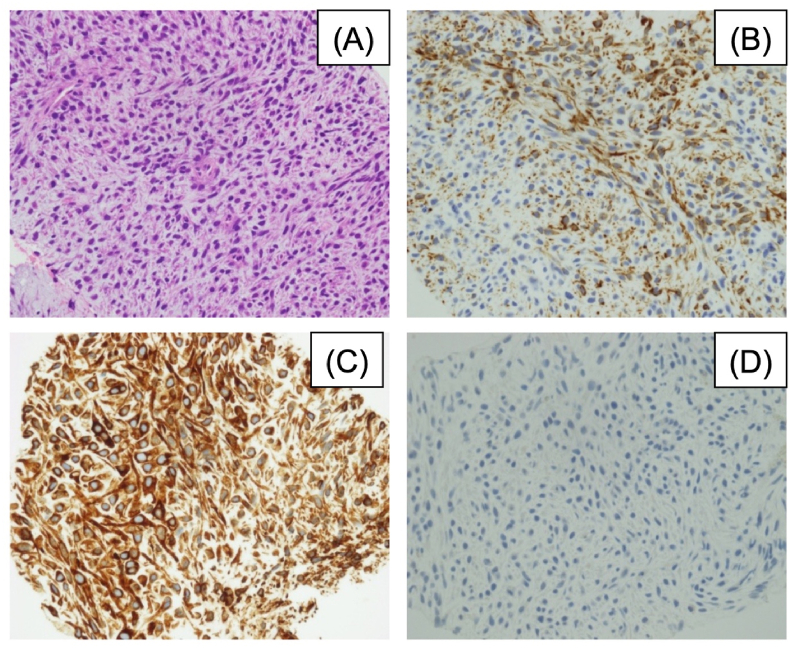
Histological images of the tumor cells. (A) Microscopic examination shows atypical spindle cells without epithelial differentiation (Haematoxylin–eosin staining; magnification ×400). Immunohistochemistry of tumor cells showing (B) CK7 (diffusely positive), (C) vimentin (diffusely positive), and (D) PD-L1(22C3) (negative).

The patient was a 50-year-old man with smoking history of 1 pack per day for 30 years and a history of viral hepatitis C. He presented fever with a body temperature of 38 °C for about a month. Chest radiography revealed a tumor shadow in the right upper lung field, and computed tomography (CT) revealed a mass measuring 14 cm and invading the mediastinum and thoracic wall in the right upper lobe of the chest along with right pleural effusion (Fig. 1A). Positron emission tomography-CT revealed abnormal tracer uptake in the area corresponding to the mass in the right upper lobe and no uptake in other organs including mediastinal lymph nodes (Fig. 2). His fever continued in the 38 °C range, although various culture tests were negative. Since there was no response to antibiotics, the patient was suspected to experience tumor fever. He underwent conventional probe endobronchial ultrasound-guided transbronchial needle aspiration (EBUS-TBNA). Histological examination revealed dense proliferation of the spindle tumor cells. Immunohistochemistry results revealed that the tumor cells were positive for CK 7 and vimentin and partially positive for AE1/AE3. Thyroid transcription factor 1 (TTF-1), napsin A, and p40 were negative in the tumor cells. PD-L1 expression (clone 22C3; Dako North America Inc.) was negative in the tumor cells (Fig. 3D). Based on the histological and immunohistochemical findings, the patient was clinically diagnosed with PSC. Malignant pleural effusion was diagnosed not cytologically but clinically because of rapid increase in pleural effusion before treatment. Therefore, he was staged as cT4N0M1a (Stage IVA). Based on the Japan Lung Cancer Society Guidelines for NSCLC [4], 2 cycles of four-agent combination chemotherapy with atezolizumab, carboplatin, paclitaxel, and bevacizumab (ABCP) were administered. Subsequently, a shrinking of the tumor size and symptomatic improvements were observed. Because of the occurrence of grade 2 peripheral sensory neuropathy and grade 2 neutropenia, according to the National Cancer Institute Common Terminology Criteria for Adverse Events, version 4.0, four-agent combination chemotherapy was completed in three cycles followed by maintenance therapy with atezolizumab and bevacizumab.

Grade 3 hypoadrenocorticism and grade 2 hypertension observed were kept well under control. Switching to maintenance therapy further caused tumor shrinkage (Fig. 1B). He completed 26 cycles of maintenance therapy, 2 years after the initiation of therapy, and the tumor shrinkage effect persisted (Fig. 1C). He had no exacerbation of symptoms and was still undergoing treatment.

## 3 Discussion

PSC is categorized as a heterogeneous group of primary lung cancers, accounting for 0.4% of NSCLC cases [1]. According to the 2015 World Health Organization (WHO) classification, PSC includes several variants of malignant epithelial tumors that histologically mimic sarcomas. PSC has been reported to be associated with poor prognosis compared to other NSCLCs. The median age of patients with PSC was 62 (54–72) years, and most of them are men (70%) and smokers (84%). They are often symptomatic (84%), and the most frequent symptoms are cough, chest pain, and weight loss [5].

Imaging studies show that peripheral tumors are more common than central tumors, and lesions are mostly localized in the upper lobe of the chest. CT images display that masses of peripheral PSCs are usually large, rounded, well-defined, often with necrotic areas, and with or without cavitation. Peripleural masses are usually subpleural and tend to invade the pleura or chest wall [6]. This 50-year-old patient was a man with a history of smoking, presenting a large subpleural tumor of 14 cm diameter in the right upper lobe, which was consistent with previous reports.

Immunostaining, in addition to cell morphology, is useful for the histological diagnosis of PSC. The differentiated epithelial component stains with cytokeratins and other markers, including epithelial membrane antigen (EMA) and carcinoembryonic antigen (CEA), whereas the sarcomatoid component often displays immunoreactivity with vimentin and fascin [7]. In particular, it has been reported that CK7 is a sensitive marker to accentuate the spindle or giant cell component and was identified in 78% of cases [8]. The histological findings of this case revealed the presence of spindle cells, which were partially positive for AE1/AE3 and positive for CK7 and vimentin, consistent with previous reports. In this case, a sufficient sample was collected using EBUS-TBNA; however, the entire tumor tissue could not be identified. Therefore, this case was classified as non-small cell carcinoma with spindle cell carcinoma. However, this patient was clinically diagnosed with PSC based on histological imaging and immunostaining findings. TTF-1 has been reported to be positive in 61% of PSC cases [9], but this case was TTF-1 negative.

No consensus in the treatment protocol for advanced PSC has been established, and there are few reports on the treatment. It has been reported that 73% of 97 patients with PSC received first-line platinum-based chemotherapy. At the first tumor evaluation, 69% of patients experienced progression, 31% had disease control, and 16.5% had a partial response. Progression-free survival (PFS) was not statistically different between the patients treated with or without platinum-based chemotherapy, although a trend towards better overall survival (OS) was noted in patients receiving platinum-based chemotherapy (7 months versus 5.3 months) [5]. In advanced non-squamous NSCLC, PFS and OS in the pemetrexed combination regimen have been reported to be significantly prolonged in the TTF-1 expression group [10]. Hence, it is difficult to positively select a pemetrexed combination regimen in TTF-1 negative PSC, similar to this case. Moreover, in the PointBreak trial [11], for histological types of non-squamous NSCLC other than adenocarcinoma and large cell carcinoma, although there was no statistically significant difference, OS and PFS tended to be longer in the group receiving carboplatin, paclitaxel, and bevacizumab (BCP) than in carboplatin, pemetrexed, and bevacizumab, suggesting that BCP is more effective on PSC than the latter.

It has been reported that the PD-L1 expression rate was higher in PSC than in other NSCLCs [2], suggesting that ICIs, such as anti-PD-1/PD-L1 antibodies can be effective treatments. ICIs have been reported to be successful against PSC with PD-L1 expression [3]; however, we could not find any report of the ICI effectiveness on PSC with no PD-L1 expression. In the IMpower 150 trial, which was the basis for applying four-agent combination chemotherapy in this case, the status of the patients with non-squamous NSCLC in groups receiving BCP or ABCP were compared [12]. In a subgroup analysis of the 0% PD-L1 expression rate group, a significant prolongation of PFS and OS was observed with ABCP treatment. However, in that study, approximately 94% of the cases were adenocarcinoma, and only 5% of “others” included PSC cases; there were no cases in the BCP group and one case in the ABCP group. Further data on the administration of PSCs are awaited.

In addition, vascular endothelial growth factor (VEGF) has been reported to be expressed in many cases of pleomorphic lung carcinoma, which is a major type of PSC [13]. This finding suggests that anti-VEGF antibodies could be effective in patients with PSC, including pleomorphic carcinoma. Preclinical studies have revealed that overexpression of VEGF makes the tumor microenvironment immunosuppressive, and antiangiogenic therapies such as anti-VEGF antibodies can rebuild the tumor microenvironment to an immunostimulatory status [14]. Therefore ABCP, especially in the phase of maintenance therapy, was extremely effective in this case, despite PD-L1 negativity because bevacizumab would have reprogrammed the microenvironment to an immunostimulatory status, and the ICI would have synergistically worked effectively against the background of VEGF overexpression in the tumor. In this case, the four-agent combination chemotherapy with ABCP was introduced, and tumor shrinkage was maintained for 2 years, suggesting that ABCP (particularly the combination of atezolizumab and bevacizumab) might be effective for advanced PSC. Although this was a single case report and it would be necessary to accumulate cases in the future, it was a very impactful case, in which chemotherapy with an ICI and antiangiogenic therapy was significantly effective, despite poor prognosis of PSC and PD-L1 being negative.

## 4 Conclusion

Our case showed that four-agent combination chemotherapy with atezolizumab, carboplatin, paclitaxel, and bevacizumab could be an effective treatment option for advanced PSC.

## Funding

The research did not receive any specific grant from funding agencies in the public, commercial, or not-for-profit sectors.

## Declaration of interests

The authors declare that they have no known competing financial interests or personal relationships that could have appeared to influence the work reported in this paper.
